# Hypoxic culture of bone marrow-derived mesenchymal stromal stem cells differentially enhances *in vitro* chondrogenesis within cell-seeded collagen and hyaluronic acid porous scaffolds

**DOI:** 10.1186/s13287-015-0075-4

**Published:** 2015-04-23

**Authors:** Troy D Bornes, Nadr M Jomha, Aillette Mulet-Sierra, Adetola B Adesida

**Affiliations:** Laboratory of Stem Cell Biology and Orthopaedic Tissue Engineering, Li Ka Shing Centre for Health Research Innovation, Divisions of Orthopaedic Surgery and Surgical Research, Department of Surgery, University of Alberta, Edmonton, AB T6G 2E1 Canada

## Abstract

**Introduction:**

The quality of cartilaginous tissue derived from bone marrow mesenchymal stromal stem cell (BMSC) transplantation has been correlated with clinical outcome. Therefore, culture conditions capable of modulating tissue phenotype, such as oxygen tension and scaffold composition, are under investigation. The objective of this study was to assess the effect of hypoxia on *in vitro* BMSC chondrogenesis within clinically approved porous scaffolds composed of collagen and hyaluronic acid (HA). It was hypothesized that hypoxic isolation/expansion and differentiation would improve BMSC chondrogenesis in each construct.

**Methods:**

Ovine BMSCs were isolated and expanded to passage 2 under hypoxia (3% oxygen) or normoxia (21% oxygen). Cell proliferation and colony-forming characteristics were assessed. BMSCs were seeded at 10 million cells per cubic centimeter on cylindrical scaffolds composed of either collagen I sponge or esterified HA non-woven mesh. Chondrogenic differentiation was performed in a defined medium under hypoxia or normoxia for 14 days. Cultured constructs were assessed for gene expression, proteoglycan staining, glycosaminoglycan (GAG) quantity, and diameter change.

**Results:**

Isolation/expansion under hypoxia resulted in faster BMSC population doublings per day (*P* <0.05), whereas cell and colony counts were not significantly different (*P* = 0.60 and 0.30, respectively). Collagen and HA scaffolds seeded with BMSCs that were isolated, expanded, and differentiated under hypoxia exhibited superior aggrecan and collagen II mRNA expressions (*P* <0.05), GAG quantity (*P* <0.05), and proteoglycan staining in comparison with normoxia. GAG/DNA was augmented with hypoxic isolation/expansion in all constructs (*P* <0.01). Comparison by scaffold composition indicated increased mRNA expressions of hyaline cartilage-associated collagen II, aggrecan, and SOX9 in collagen scaffolds, although expression of collagen X, which is related to hypertrophic cartilage, was also elevated (*P* <0.05). Proteoglycan deposition was not significantly improved in collagen scaffolds unless culture involved normoxic isolation/expansion followed by hypoxic differentiation. During chondrogenesis, collagen-based constructs progressively contracted to 60.1% ± 8.9% of the initial diameter after 14 days, whereas HA-based construct size was maintained (109.7% ± 4.2%).

**Conclusions:**

Hypoxic isolation/expansion and differentiation enhance *in vitro* BMSC chondrogenesis within porous scaffolds. Although both collagen I and HA scaffolds support the creation of hyaline-like cartilaginous tissue, variations in gene expression, extracellular matrix formation, and construct size occur during chondrogenesis.

**Electronic supplementary material:**

The online version of this article (doi:10.1186/s13287-015-0075-4) contains supplementary material, which is available to authorized users.

## Introduction

Bone marrow-derived mesenchymal stromal stem cells (BMSCs) are a promising cell-based option for treating articular cartilage defects [1-6]. Clinical and pre-clinical studies have shown variable outcomes following BMSC transplantation for treatment of focal chondral and osteochondral defects [7]. Repair tissues consistent with hyaline cartilage, fibrocartilage, and mixed tissue have been reported [2-4]. Clinical scores correlate with quality of cartilaginous repair tissue on the basis of magnetic resonance imaging and histological analysis [2,4,6]. Therefore, culture conditions capable of improving cell and tissue phenotype are currently under investigation.

Incubator oxygen tension is a culture variable that has gained attention on the basis of the posited role of oxygen in musculoskeletal tissue development and cellular microenvironments. There is evidence to suggest that hypoxia promotes chondrogenic differentiation of BMSCs during pre-natal limb development [8]. Furthermore, BMSCs exist in hypoxic bone marrow spaces, whereas chondrocytes reside within avascular hyaline cartilage and are bathed in hypoxic synovial fluid [9,10].

The positive impact of hypoxia on BMSC proliferation has been demonstrated on the basis of cell count, nucleoside incorporation, and colony-forming capability [11-15]. During prolonged expansion periods, stem cell characteristics such as rapid proliferation and multipotency are maintained with hypoxic incubation [11,12], whereas senescence is delayed [16]. Hypoxic BMSC isolation and expansion [13-15,17,18] and hypoxic BMSC differentiation [12-15,17,19] have separately been associated with improved *in vitro* chondrogenesis within pellet, micromass, and hydrogel models. Three studies have compared the impact of hypoxic isolation/expansion with hypoxic differentiation on chondrogenesis, and variable improvements in gene expression and cartilaginous extracellular matrix (ECM) formation were found with hypoxic exposure during each distinct culture period [14,15,19].

Although hypoxic enhancement of BMSC chondrogenesis has been studied extensively in pellet, micromass, and hydrogel models, this effect has not been elucidated in detail in porous scaffolds. Porous scaffolds composed of natural and synthetic materials allow cells to permeate, adhere, and organize within a three-dimensional (3D) environment, and deposit ECM to form tissue [20]. As a result, they serve as a suitable model for studying 3D cartilage formation *in vitro* [7]. Moreover, porous scaffolds composed of collagen or hyaluronic acid (HA) are commonly used in clinical BMSC transplantation [2-5,21]. At present, it is not known whether hypoxic culture improves chondrogenesis of BMSCs seeded on 3D porous scaffolds. Accordingly, the first objective of this study was to assess the effect of oxygen tension during distinct isolation/expansion and differentiation culture periods on chondrogenesis within BMSC-seeded porous scaffolds. The impact of porous scaffold material on the modulation of chondrogenesis with oxygen tension has not been elucidated. Therefore, the second objective of this study was to investigate differences in chondrogenesis between BMSCs seeded and cultured on a collagen I porous scaffold and an esterified HA porous scaffold. It was hypothesized that hypoxic incubation during isolation/expansion and differentiation culture periods would improve BMSC chondrogenesis within each scaffold.

## Methods

### Bone marrow aspiration and mononucleated cell counting

Bone marrow-derived cell collections for this study were obtained from iliac crest aspirates from six skeletally mature, female Suffolk sheep (mean age ± standard error of the mean (SEM) of 3.3 ± 0.8 years). Characteristics of each sheep are summarized in Table 1. General anesthesia for the aspiration procedure was attained through sedation with intravenous dexmedetomidine (5 μg/kg) and ketamine (2 mg/kg) followed by endotracheal intubation and administration of gaseous isoflurane (2% to 4% in oxygen). The sheep were then positioned in a lateral decubitus position, and the posterolateral pelvic surgical site was clipped and prepared with 10% wt/vol povidone-iodine solution (Betadine; Purdue Pharma L.P., Stamford, CT, USA). A small incision was made over the posterior ilium, and an 11-gauge Jamshidi needle (Cardinal Health Canada Inc., Vaughan, ON, Canada) was inserted onto the iliac crest near the posterior superior iliac spine and through cortical bone into the marrow space. Bone marrow aspirate (mean volume ± SEM of 33 ± 2 mL) was collected and mixed immediately with 8 mL of heparin (10,000 units per 10 mL; Pharmaceutical Partners of Canada Inc., Richmond Hill, ON, Canada). Aspirates were then filtered with a cell strainer (100-μm pore size; Becton Dickinson Canada Inc., Mississauga, ON, Canada). The number of mononucleated cells (MNCs) in each aspirate was determined by crystal violet nuclei staining and cell counting by using a hemocytometer.

**Table 1:** **Bone marrow donor information**

**Donor**	**Gender**	**Age, years**	**Mass, kilograms**
Z28	Female	2.0	66
Z01	Female	2.2	71
Z33	Female	2.3	81
Y19	Female	3.2	63
Y08	Female	3.3	94
T10	Female	7.0	74

### Isolation and expansion of bone marrow-derived mesenchymal stem cells

Bone marrow aspirate collections containing 8 × 10^7^ MNCs were seeded within each 150-cm^2^ tissue culture flask. Culture medium composed of alpha-minimal essential medium (α-MEM) supplemented with 10% vol/vol heat-inactivated fetal bovine serum (FBS), penicillin-streptomycin-glutamine, 4-(2-hydroxyethyl)-1-piperazineethanesulfonic acid (HEPES), and sodium pyruvate (all from Life Technologies, Burlington, ON, Canada) was pipetted into each flask. Fibroblast growth factor-2 (FGF-2) (Neuromics Inc., Edina, MN, USA) was added at a concentration of 5 ng/mL in order to maintain cell multipotency [22]. Nucleated cells were allowed to adhere and grow for 7 days before the first media change under normoxia (ambient 21% O_2_) or hypoxia (low 3% O_2_) at 37°C in a humidified incubator containing 5% CO_2_. Flasks from the hypoxic incubator experienced short periods (<5 minutes) of normoxic exposure during media changes. Thereafter, the media were changed twice per week until 80% cell confluence was obtained. Adherent BMSCs were detached by using 0.05% wt/vol trypsin-ethylenediaminetetraacetic acid (EDTA) (Sigma-Aldrich, Oakville, ON, Canada) and expanded under the same oxygen tension (normoxia or hypoxia) as during isolation until passage 2 (P2) prior to scaffold seeding. Hereafter for brevity, BMSCs described by expansion oxygen tension alone (normoxia-expanded and hypoxia-expanded BMSCs) will refer to BMSCs that were isolated and expanded under normoxia and hypoxia, respectively. The time taken from plating of nucleated cells (P0) to reach approximately 80% confluence at P2, before experimental use, varied from 3 to 4 weeks.

### Colony-forming unit fibroblastic assay

A colony-forming unit fibroblastic (CFU-F) assay was conducted to determine the effect of oxygen tension on colony-forming characteristics of BMSCs and to calculate the proportion of plastic-adherent cells (BMSCs) derived from bone marrow aspirates containing a known number of MNCs. MNCs from each donor were plated in triplicate at 1 × 10^5^ MNCs per 100-mm-diameter sterile petri dish (Becton Dickinson Canada Inc.) and cultured as described for expansion conditions under normoxia (21% O_2_) or hypoxia (3% O_2_) by using α-MEM supplemented with 10% vol/vol heat-inactivated FBS, penicillin-streptomycin, HEPES, sodium pyruvate, and 5 ng/mL FGF-2. After the first week, the non-adherent cell population was removed by aspiration and culture media were replenished twice each week. During media changes, hypoxia-cultivated cells experienced a short period (<5 minutes) of exposure to normoxia. The total duration of culture time used for each donor (mean ± SEM of 16.3 ± 0.5 days) was equivalent to the time required to attain 80% BMSC confluence at P0 and subsequent detachment and splitting to P1 for expansion in T150 flasks (see ‘Isolation and expansion of bone marrow-derived mesenchymal stem cells’ section). For each donor, the culture times for petri dishes placed in hypoxic and normoxic conditions were identical. After the CFU-F culture period finished, the petri dishes were fixed with 10% wt/vol buffered formalin (3.8% wt/vol formaldehyde), washed by using phosphate-buffered saline (PBS) (Life Technologies), and stained with 0.25% wt/vol crystal violet solution (Sigma-Aldrich). During analysis, each stained cell collection was assessed and considered to be a colony only if (a) the cell collection was stained strongly with crystal violet, (b) the periphery of the collection was circular and well defined with respect to the surrounding area of the plate that was not stained, and (c) the diameter of the stained cell collection was measured to be at least 2 mm. The number of BMSC colonies in each dish was recorded, as was the diameter of each colony.

### Bone marrow-derived mesenchymal stem cell counts and doubling

Total cell counts of trypsinized BMSCs at P0, P1, and P2 were calculated by using trypan blue staining and hemocytometer counting of small aliquots of BMSCs in expansion medium. The number of colonies noted in each CFU-F assay was used to determine the number of BMSCs isolated per 1 × 10^5^ MNCs plated for each donor, and this was extrapolated to calculate the number of BMSCs arising from 8 × 10^7^ MNCs plated in each T150 culture flask during P0 expansion. Population doublings were determined by using the method described by Solchaga *et al*. [22].

### Trilineage differentiation potential of bone marrow-derived mesenchymal stem cells

Osteogenic differentiation was performed by plating 5 × 10^3^ BMSCs/cm^2^ within wells of a six-well plate (Becton Dickinson Canada Inc.) and culturing these in 2.5 mL of osteogenic medium for 21 days at 37°C in a humidified hypoxic incubator (3% O_2_ and 5% CO_2_). Osteogenic medium consisted of Dulbecco’s modified Eagle’s medium (DMEM) containing 4.5 mg/mL D-glucose, 0.1 mM non-essential amino acids, 1 mM sodium pyruvate, 100 mM HEPES, 100 U/mL penicillin, 100 μg/mL streptomycin, 0.29 mg/mL L-glutamine (all from Life Technologies) supplemented with 10% vol/vol heat-inactivated FBS, 0.1 mM ascorbic acid 2-phosphate, 10 nM dexamethasone, and 10 mM β-glycerophosphate (Sigma-Aldrich). Media changes were performed twice per week. After the culture period, the contents of each well were fixed with 10% wt/vol buffered formalin (3.8% wt/vol formaldehyde), stained for 30 minutes with 1% wt/vol Alizarin Red S (Sigma-Aldrich) with an adjusted pH of 4.2, washed with distilled water over 60 minutes, and stored at 4°C in 70% vol/vol glycerol (Fisher Scientific Chemical Division, Fair Lawn, NJ, USA). Images were captured by using an Omano OM159T biological trinocular microscope (Microscope Store, Roanoke, VA, USA) fitted with an Optixcam Summit Series 5MP digital camera and Optixcam software and assembled in Photoshop (Adobe Systems Inc., San Jose, CA, USA).

Adipogenic differentiation was performed on BMSCs plated at 5 × 10^3^ BMSCs/cm^2^ within wells of a six-well plate (Becton Dickinson Canada Inc.). Plated BMSCs were initially cultured in basic culture medium of 2.5 mL of DMEM containing 4.5 mg/mL D-glucose, 0.1 mM non-essential amino acids, 1 mM sodium pyruvate, 100 mM HEPES, 100 U/mL penicillin, 100 μg/mL streptomycin, and 0.29 mg/mL L-glutamine supplemented with 10% vol/vol heat-inactivated FBS (all from Life Technologies). Adipogenesis was then induced over the course of 3 days in 2.5 mL of basic culture medium supplemented with 1 μM dexamethasone, 0.5 mL insulin-transferrin-selenium (ITS) + 1, 100 μM indomethacin, and 500 μM isobutyl-1-methylxanthine (IBMX) (Sigma-Aldrich), followed by 1 day of culture in 2.5 mL of basic culture medium supplemented with only 0.5 mL ITS + 1. This 4-day cycle was repeated three times, followed by culture for 7 days in 2.5 mL of basic culture medium supplemented with only 0.5 mL ITS + 1. All culture was performed in a hypoxic humidified incubator (3% O_2_ and 5% CO_2_). After the culture period, the contents of each well were fixed with 10% wt/vol buffered formalin (3.8% wt/vol formaldehyde), stained for 60 minutes with 0.3% wt/vol Oil Red O (Sigma-Aldrich), washed with distilled water, and assessed immediately for staining. Oil Red O solution was prepared by creating a 0.5% wt/vol solution of Oil Red O (Sigma-Aldrich) in isopropanol (Fisher Scientific Chemical Division) and then diluting this to 0.3% wt/vol in an equivalent volume of distilled water. Microscopic images were captured in an identical fashion to osteogenic culture products.

Chondrogenic differentiation was verified by using a pellet culture system. BMSCs were suspended in chondrogenic medium consisting of DMEM containing 4.5 mg/mL D-glucose, 0.1 mM non-essential amino acids, 1 mM sodium pyruvate, 100 mM HEPES, 100 U/mL penicillin, 100 μg/mL streptomycin, 0.29 mg/mL L-glutamine (all from Life Technologies) supplemented with 0.1 mM ascorbic acid 2-phosphate, 0.1 μM dexamethasone, 1x ITS + 1 premix (Sigma-Aldrich), and 10 ng/mL transforming growth factor-beta 3 (TGF-β3) (Neuromics Inc.). In total, 5 × 10^5^ BMSCs were spun within 1.5-mL sterile conical polypropylene microfuge tubes (Enzymax LLC, Lexington, KY, USA) at 1,500 revolutions per minute for 5 minutes to form spherical cell pellets. Pellets were then submersed in 500 μL of chondrogenic medium and cultured in a hypoxic humidified incubator (3% O_2_ and 5% CO_2_) for 21 days. Pellets experienced a short period (<5 minutes) of exposure to normal oxygen tension during media changes. After the culture period, BMSC pellets were processed and stained for histological analysis of cartilaginous proteoglycans (see ‘Histological and immunohistochemical analyses of extracellular matrix contents’ section).

### Porous scaffold seeding and chondrogenic differentiation of bone marrow-derived mesenchymal stem cell-seeded scaffolds

Cylindrical collagen scaffolds (3.5-mm thickness and 6-mm diameter) were created by using a biopsy punch on sheets of porous, type I collagen sponge (125 × 100 × 3.5 mm^3^ dimension and 115 ± 20 μm pore size; Integra LifeSciences Corp., Plainsboro Township, NJ, USA). Cylindrical HA scaffolds (2-mm thickness and 6-mm diameter) were created by using a biopsy punch on sheets of esterified HA (HYAFF) non-woven mesh (20 × 20 × 2 mm^3^ dimensions and 10- to 20-μm-diameter fibers with varying inter-fiber spaces [23]; Anika Therapeutics S.r.l., Abano Terme, PD, Italy). Scaffold seeding was performed at a consistent density of 1 × 10^7^ BMSCs/cm^3^, which corresponded to 989,602 BMSCs seeded per collagen scaffold and 565,487 BMSCs seeded per HA scaffold [24].

During seeding, BMSCs at P2 were re-suspended in chondrogenic culture medium (see ‘Trilineage differentiation potential of bone marrow-derived mesenchymal stem cells’ section). Total cell counts were calculated from trypan blue staining and hemocytometer counting of small aliquots of BMSCs. BMSCs were micropipetted onto each scaffold within a 20-μL chondrogenic medium suspension. HA scaffolds were pre-soaked with 20 μL of chondrogenic medium to promote dispersion of the cell suspension over the scaffold during seeding. Seeded scaffolds were then incubated at 37°C for 15 minutes followed by the addition of 100 μL of chondrogenic medium to the base of each scaffold. Thereafter, constructs were incubated for an additional 30 minutes to promote cell adhesion. All constructs, including cell-free scaffolds (control group) and those seeded with either normoxia- or hypoxia-expanded BMSCs, were subsequently immersed in 1 mL of chondrogenic medium and cultured statically at 37°C within humidified incubators containing 5% CO_2_ and either 21% O_2_ (normoxia) or 3% O_2_ (hypoxia). Media were changed twice weekly thereafter.

### Oxygen tension experimental groups

**Figure 1:**
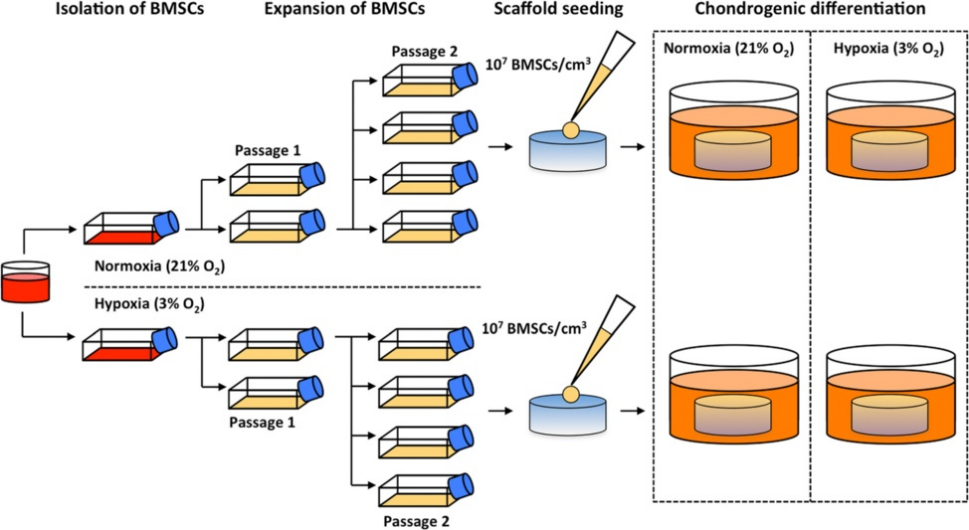
*In vitro* cartilage engineering from bone marrow-derived mesenchymal stem cell (BMSC)-seeded porous scaffolds. BMSCs were isolated by plastic adherence from bone marrow aspirates and expanded in tissue culture flasks to passage 2 within defined expansion medium containing fetal bovine serum and fibroblast growth factor-2 under either normoxic (21% O_2_) or hypoxic (3% O_2_) incubator conditions. Thereafter, BMSCs were seeded at 10 million cells per cubic centimeter onto clinically approved, cylindrical, porous scaffolds composed of collagen or hyaluronic acid. BMSC-scaffold constructs were subsequently cultured under either normoxia or hypoxia for 14 days within serum-free chondrogenic medium containing transforming growth factor-beta 3 and dexamethasone.

To determine the effect of oxygen tension during isolation/expansion and differentiation on *in vitro* chondrogenesis within BMSC-seeded scaffolds, BMSCs from five donors (Z28, Z01, Z33, Y19, and T10) with a mean age (± SEM) of 3.3 ± 0.9 years were seeded onto collagen and HA scaffolds and cultured in chondrogenic medium for 14 days. The experimental set-up is illustrated in Figure 1. The treatment groups included the following: Hyp/Hyp (isolation and expansion under hypoxia and differentiation under hypoxia), Hyp/Nrx (isolation and expansion under hypoxia and differentiation under normoxia), Nrx/Hyp (isolation and expansion under normoxia and differentiation under hypoxia), and Nrx/Nrx (isolation and expansion under normoxia and differentiation under normoxia). After culture, the tissue-engineered constructs were assessed with reverse transcription-quantitative real-time polymerase chain reaction (RT-qPCR) for gene expression, histological staining of ECM proteoglycans, collagen II immunohistochemistry, and biochemical quantification of glycosaminoglycan (GAG) and DNA.

### Reverse transcription-quantitative real-time polymerase chain reaction analysis of chondrogenic genes

Total RNA was extracted from constructs by using TRIzol Reagent (Life Technologies) after grinding with a pestle. To mitigate changes in gene expression, constructs under hypoxia and normoxia were immediately transferred into TRIzol Reagent following removal from respective incubators. Total RNA (100 ng) in a 40-μL reaction was reverse-transcribed to cDNA by using a GoScript Reverse Transcription System (Promega Corporation, Madison, WI, USA) primed in the presence of oligo(dT) primers (1 μg). Quantitative polymerase chain reaction was performed with a DNA Engine Opticon II Continuous Fluorescence Detection System (Bio-Rad Laboratories, Hercules, CA, USA) by using HotGoldStar Taq polymerase and SYBR Green detection (Eurogentec North America Inc., San Diego, CA, USA). Primer sequences (Table 2) were created by using information from the National Center for Biotechnology Information (NCBI) database and custom-designed by using the Primer Express software (Applied Biosystems, Foster City, CA, USA). All primers were obtained from Invitrogen (Life Technologies). Gene (mRNA) expression levels for each primer set were normalized to the expression level of ovine β-actin by the 2^−ΔΔC(T)^ method [25].

**Table 2:** **Ovine primer sequences used in reverse transcription-quantitative real-time polymerase chain reaction analysis**

**Gene**	**Primer sequences**		**NCBI reference**
β-actin (*ACTB*)	5′-CGGCGGGACCACCAT-3′	Forward	NM_001009784.1
	5′-GCAGTGATCTCTTTCTGCATCCT-3′	Reverse	
Aggrecan (*ACAN*)	5′-TGGAATGATGTCCCATGCAA-3′	Forward	XM_004018048.1
	5′-GCCACTGTGCCCTTTTTACAG-3′	Reverse	
COMP (*COMP*)	5′-CCTAACTGGGTGGTGCTCAAC-3′	Forward	XM_004009150.1
	5′-CTGGGTCGCTGTTCATCGT-3′	Reverse	
Collagen I (*COL1A1*)	5′-CGCCCCAGACCAGGAATT-3′	Forward	XM_004012773.1
	5′-GTGGAAGGAGTTTACAGGAAGCA-3′	Reverse	
Collagen II (*COL2A1*)	5′-GACCTCACGTCTCCCCATCA-3′	Forward	XM_004006408.1
	5′-CTGCTCGGGCCCTCCTAT-3′	Reverse	
Collagen X (*COL10A1*)	5′-CAGGCTCGAATGGGCTGTAC-3′	Forward	XM_004011185.1
	5′-CCACCAAGAATCCTGAGAAAGAG-3′	Reverse	
SRY-Box 9 (*SOX9*)	5′-GCTGCTGGCCGTGATGA-3′	Forward	XM_004013527.1
	5′-GGGTCGCGCGTTTGTTT-3′	Reverse	

NCBI, National Center for Biotechnology Information.

RT-qPCR products were subsequently sequenced to confirm that expression levels were based on appropriate sequences for each gene. For sequencing, RT-qPCR products were purified by using a QIAquick Gel Extraction Kit (Qiagen Inc. Canada, Toronto, ON, Canada), combined with each primer (forward and reverse primers separately), and sequenced by using the Sanger method (The Applied Genomics Centre, University of Alberta, Edmonton, AB, Canada). The resulting traces were read by using FinchTV Software Version 1.5 (Geospiza Inc., Seattle, WA, USA) and matched to corresponding regions within characterized gene sequences by using the nucleotide Basic Local Alignment Search Tool (BLAST) (NCBI). Sequencing analysis is included within Additional file 1.

### Histological and immunohistochemical analyses of extracellular matrix contents

Pellets and scaffold-based constructs were removed from media, fixed in 10% wt/vol buffered formalin (3.8% wt/vol formaldehyde), processed into paraffin wax, and sectioned at a thickness of 5 μm. Sections were stained with 0.1% wt/vol safranin O to reveal proteoglycan matrix depositions and counterstained with 1% wt/vol fast green. Other sections from scaffold-based constructs were treated with chondroitinase (Sigma-Aldrich) at 0.2 U per section and incubated with an antibody against collagen II (II-II6B3 from Developmental Studies Hybridoma Bank at the University of Iowa, Iowa City, IA, USA; 1:50 dilution). Immune-localized antigens were visualized with horse anti-mouse IgG biotinylated secondary antibody (Vectastain; Vector Laboratories Inc., Burlingame, CA, USA) and an aminoethylcarbazole (AEC)-based peroxidase labeling kit (Enzo Life Sciences Inc., Farmingdale, NY, USA). Images were captured by using an Eclipse Ti-S microscope (Nikon Canada Inc., Mississauga, ON, Canada) fitted with NIS Elements Basic Research Imaging Software Version 4.20 (Nikon Canada Inc.) and assembled in Photoshop.

### Biochemical analysis of glycosaminoglycan and DNA quantity

*In vitro* cultured BMSC-scaffold constructs were rinsed in PBS and digested in proteinase K (1 mg/mL in 50 mM Tris with 1 mM EDTA, 1 mM iodoacetamide, and 10 mg/mL pepstatin A; all from Sigma-Aldrich) for 16 hours at 56°C. Sulfated GAG content was measured by 1,9-dimethylmethylene blue binding (Sigma-Aldrich) by using chondroitin sulfate (Sigma-Aldrich) as the standard. DNA content was determined by using the CyQUANT Cell Proliferation Assay Kit (Life Technologies) with supplied bacteriophage λ DNA as the standard.

### Analysis of bone marrow-derived mesenchymal stem cell-scaffold construct size

To characterize changes in diameter with culture time, BMSC-seeded scaffolds (five per donor) were photographed with a high-quality digital camera after 7 and 14 days of *in vitro* chondrogenic culture. Each image was evaluated for diameter changes as previously described [26,27]. The diameter of each scaffold was measured in four separate planes with ImageJ software (National Institutes of Health, Bethesda, MD, USA), and the resulting mean diameter was expressed as a percentage change from the initial diameter at the time of seeding [26].

### Statistical analysis

Analyses were performed by using SPSS Statistics 22 (IBM Corporation, Armonk, NY, USA), and a *P* value of less than 0.05 was considered significant. Proliferation analyses used a repeated measures analysis of variance (ANOVA) for BMSC count and doublings over time, and a *t* test was used for assessment of these parameters on specific culture days. CFU-F analysis involved a Mann-Whitney *U* test for assessment of colony count and diameter. For analyses of gene expression and biochemical quantities, a Kruskal-Wallis one-way ANOVA was used to determine differences between the four experimental groups with pairwise (*post hoc*) comparisons, and a Mann-Whitney *U* test was used for pooled analyses. For analysis of construct diameter, a repeated measures ANOVA was used to determine differences between the four experimental groups over time, and a *t* test was used for pooled analysis at each time point.

### Ethical considerations

All experiments were implemented with BMSCs isolated from bone marrow aspirates taken from adult sheep after ethical approval from the University of Alberta’s Animal Care and Use Committee.

## Results

### Hypoxic isolation and expansion to passage 2 may enhance bone marrow-derived mesenchymal stem cell proliferation

**Figure 2:**
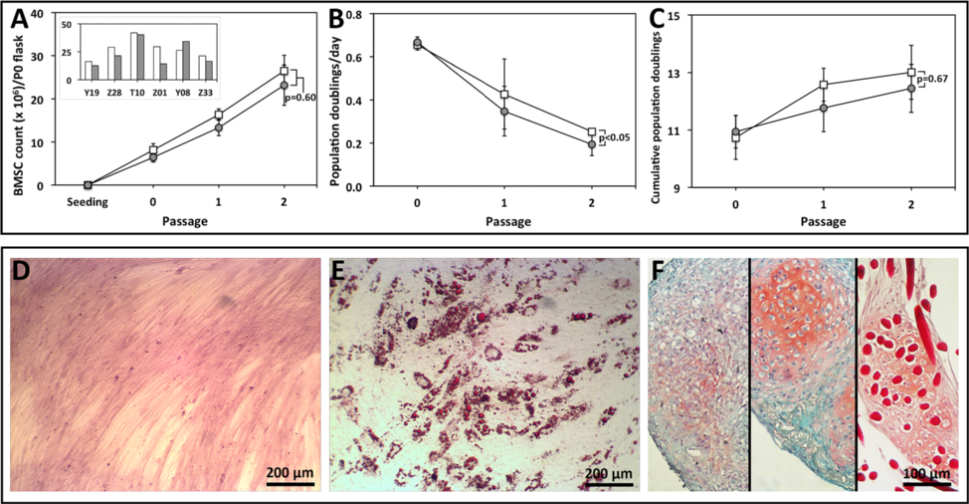
Expansion and trilineage differentiation of bone marrow-derived mesenchymal stem cells (BMSCs). **(A)** Cell counts, **(B)** population doublings per day, and **(C)** cumulative population doublings at each passage during expansion of BMSCs under normoxia or hypoxia. Data points represent mean ± standard error of the mean of cells from six donors, and *P* values are listed. **(D)** Osteogenic differentiation of BMSCs verified with Alizarin Red S staining following expansion under hypoxia and monolayer culture within medium containing β-glycerophosphate, dexamethasone, and fetal bovine serum. **(E)** Adipogenic differentiation of BMSCs verified with Oil Red O staining following expansion under hypoxia and monolayer culture within medium containing isobutyl-1-methylxanthine (IBMX), indomethacin, dexamethasone, and fetal bovine serum. **(F)** Chondrogenic differentiation of BMSCs verified by safranin O staining following expansion under hypoxia and culture performed in pellets (left) or scaffolds composed of collagen (middle) or hyaluronic acid (right) submersed in a defined serum-free chondrogenic medium containing transforming growth factor-beta 3 and dexamethasone.

Similar cell counts were found for hypoxia- and normoxia-expanded BMSCs throughout the expansion period (*P* = 0.60, Figure 2A). At the end of P2, there were 26.5 ± 3.6 million and 23.2 ± 4.7 million BMSCs yielded from each T150 flask plated at P0 and expanded under hypoxia and normoxia, respectively. Five of six donors had higher BMSC counts under hypoxia (Figure 2A inset). However, mean values were not statistically different (*P* = 0.74). Population doublings per day were highest at P0 and decreased significantly with subsequent passages (*P* = 0.02; Figure 2B). Hypoxia-expanded BMSCs had higher population doublings per day than normoxia-expanded BMSCs throughout the expansion period (*P* <0.05). Cumulative population doublings were not different between groups (*P* = 0.67; Figure 2C). Values of mean time (± SEM) between plating at P0 and 80% BMSC confluence at the end of P2 were 23.4 ± 0.8 days for hypoxia-expanded BMSCs and 25.3 ± 1.5 days for normoxia-expanded BMSCs (*P* = 0.38).

**Figure 3:**
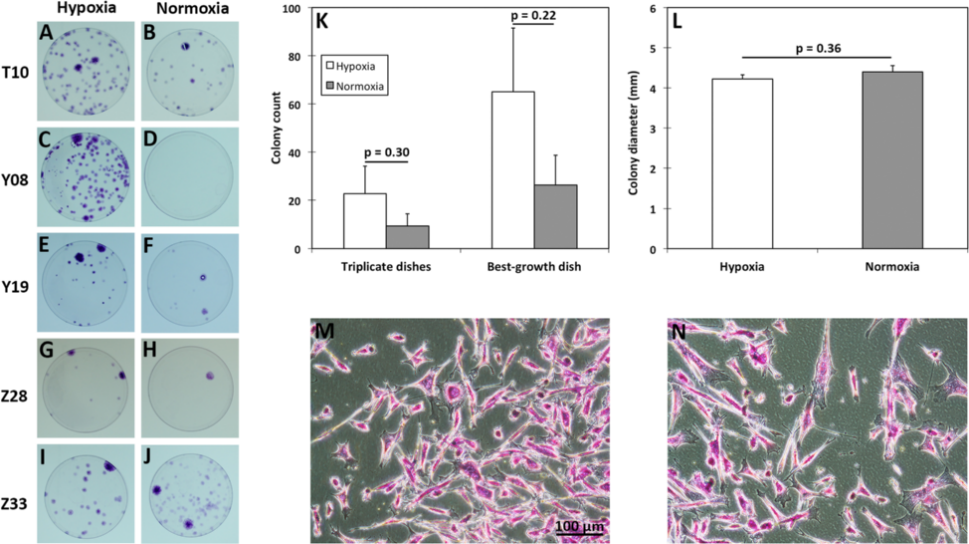
Colony-forming unit fibroblastic assay of passage 0 bone marrow-derived mesenchymal stem cells isolated and expanded under normoxia or hypoxia. **(A**-**J)** Plastic-adherent bone marrow-derived mesenchymal stem cells were isolated and expanded under hypoxia or normoxia and formed colonies that were stained with crystal violet for visualization. Dishes seeded with cells from donor Z01 were excluded because of a lack of colony formation. **(K**, **L)** Colony counts and diameters were measured and are reported as mean ± standard error of the mean, and *P* values are listed. Representative photomicrographs of adherent cells from donor Y19 formed under hypoxic **(M)** and normoxic **(N)** culture.

A CFU-F assay was performed to determine the effect of oxygen tension on colony-forming characteristics of adherent BMSCs isolated from plated MNCs and cultured in expansion medium under normoxia and hypoxia. Colony counts and diameters were assessed at the end of P0 when corresponding BMSCs undergoing expansion in T150 flasks had reached 80% confluence (16.3 ± 0.5 days of culture). Seeded petri dishes from one donor (Z01) lacked colony formation over this period. Hypoxic isolation/expansion augmented BMSC colony counts in petri dishes seeded with cells from four of the remaining five donors (Figure 3A-J), although there was not a significant difference in mean colony counts between the groups (*P* = 0.30 for triplicate dishes and *P* = 0.22 for best-growth dishes; Figure 3K and L). Crystal violet staining of adherent cells in hypoxia- and normoxia-cultured dishes showed the presence of a spectrum of cell morphologies ranging from long, spindle-shaped cells to circular and cuboidal cells (Figure 3M and N). No difference was noted in colony diameter between culture groups (*P* = 0.36; Figure 3L).

### Trilineage differentiation of adherent bone marrow-derived mesenchymal stem cells

Plastic-adherent BMSCs were expanded to P2 and differentiated *in vitro* toward bone, adipose, and cartilage tissue lineages to confirm multilineage potential [28]. Tissue derived from BMSCs stained extensively with Alizarin Red S (Figure 2D) and Oil Red O (Figure 2E), which confirmed the presence of bone and adipose matrix following culture in defined osteogenic and adipogenic media, respectively. Positive safranin O staining of sections from BMSC pellets and cell-seeded scaffolds following culture in chondrogenic medium supported the chondrogenic capacity of the adherent cells (Figure 2F).

### Hypoxic isolation/expansion and differentiation augment chondrogenic gene expression differentially within bone marrow-derived mesenchymal stem cell-seeded collagen and hyaluronic acid scaffolds

**Figure 4:**
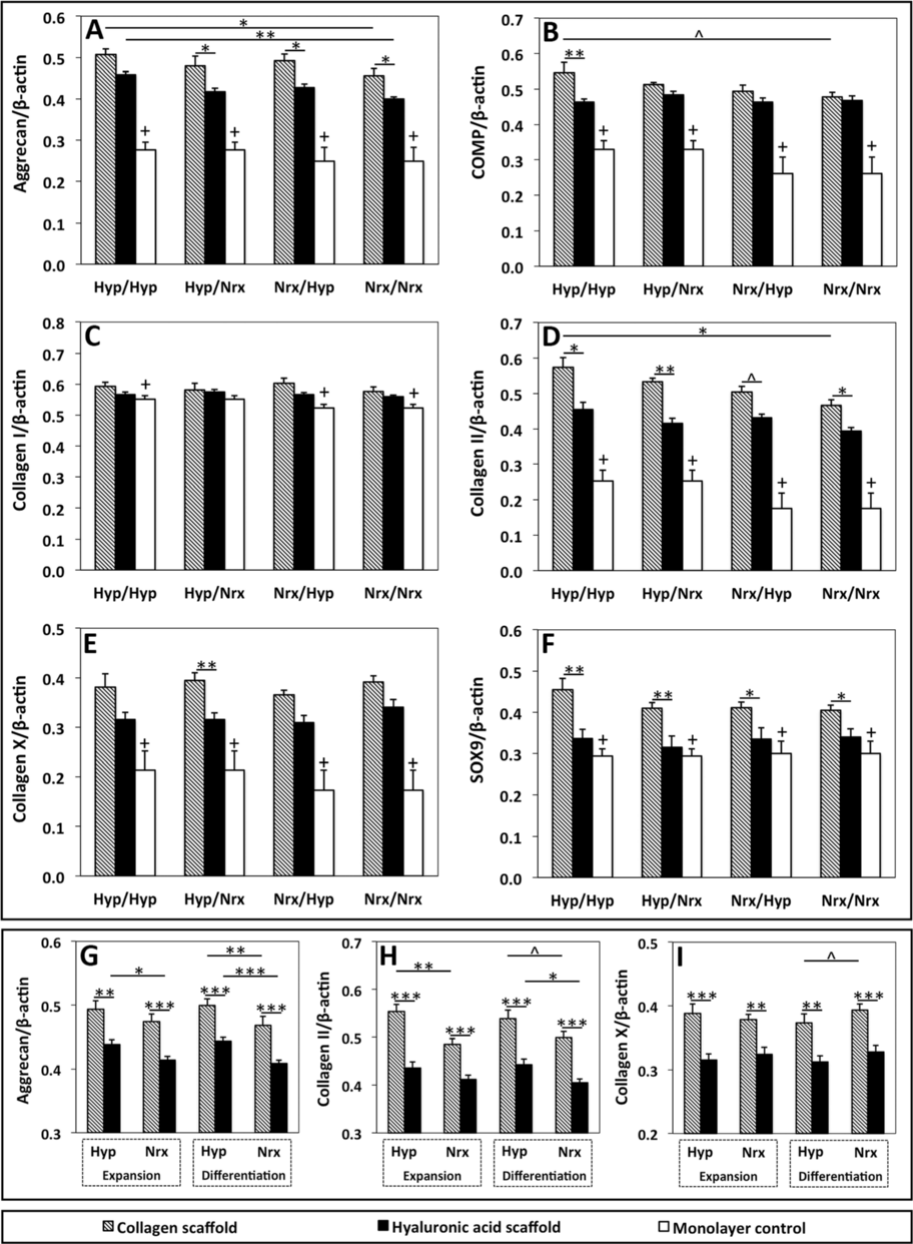
Reverse transcription-quantitative real-time polymerase chain reaction (RT-qPCR) analysis of gene expression within collagen and hyaluronic acid (HA) scaffolds seeded with hypoxia- and normoxia-cultured bone marrow-derived mesenchymal stem cells (BMSCs). BMSCs were isolated/expanded under normoxia or hypoxia, seeded within collagen or HA scaffolds, and subsequently differentiated under normoxia or hypoxia for 14 days in chondrogenic medium. RT-qPCR was performed by using SYBR Green detection. **(A-F)** Data represent the mean ± standard error of the mean (SEM) of constructs in doublets based on oxygen tension group. **(G-I)** Data represent mean ± SEM of constructs from the same donors pooled on the basis of oxygen tension during isolation/expansion or differentiation. Statistical analysis is represented by the following: unlabeled indicates not significant, ^approaching significance *P* = 0.05-0.10, *significant *P* <0.05, **significant *P* <0.01, ***significant *P* <0.001, and ^+^monolayer control value that is significantly different than BMSC-seeded scaffolds (*P* <0.05). COMP, cartilage oligomeric matrix protein; SOX9, sex-determining region Y (SRY)-box 9.

The impact of oxygen tension during isolation/expansion and differentiation on BMSC chondrogenic gene expression within collagen and HA scaffolds was assessed with RT-qPCR after 14 days of chondrogenic culture. For BMSC-seeded collagen scaffolds, Hyp/Hyp constructs had significantly higher aggrecan and collagen II mRNA expressions than Nrx/Nrx constructs (*P* <0.05; Figure 4A and D), and a trend was seen for cartilage oligomeric matrix protein (COMP) and collagen II/I ratio (*P* = 0.06 and 0.09, respectively; Figure 4B). Collagen I, collagen X, and sex-determining region Y (SRY)-box 9 (SOX9) expressions and the ratio of collagen II/X were not significantly different between oxygen tension groups (*P* = 0.20, 0.31, 0.22, and 0.40, respectively; Figure 4C, E, and F). Within HA scaffolds, Hyp/Hyp BMSCs had significantly higher aggrecan mRNA expression than Nrx/Nrx BMSCs (*P* = 0.009; Figure 4A). COMP, collagen I, collagen II, collagen X, and SOX9 gene expressions and the ratios of collagen II/I and collagen II/X were not significantly different between oxygen tension groups (*P* = 0.72, 0.60, 0.13, 0.32, 0.99, 0.17, and 0.40, respectively; Figure 4B-F).

To further characterize the impact of oxygen tension on gene expression, data were pooled on the basis of expansion oxygen tension and differentiation oxygen tension. BMSCs expanded under hypoxic conditions and seeded within collagen scaffolds produced significantly higher mRNA expression of collagen II and increased ratios of collagen II/I and collagen II/X than BMSCs expanded under normoxic conditions (*P* <0.01; Figure 4H). However, aggrecan expression was not significantly different between groups (*P* = 0.19; Figure 4G). HA scaffolds seeded with BMSCs expanded under hypoxia produced significantly higher mRNA expression of aggrecan than BMSCs expanded under normoxia (*P* = 0.02; Figure 4G). Collagen II mRNA expression and ratios of collagen II/I and collagen II/X were not significantly different (*P* = 0.31 and 0.74, respectively; Figure 4H). Within both collagen- and HA-based constructs, oxygen tension during expansion did not significantly impact collagen I, collagen X, COMP, or SOX9 mRNA expression (*P* ≥0.29, 0.52, 0.14, and 0.16, respectively).

Pooling of data on the basis of differentiation condition indicated that hypoxic differentiation also augmented chondrogenic gene expression. Collagen scaffolds seeded with BMSCs and differentiated under hypoxia had significantly higher aggrecan and collagen I mRNA expressions (*P* = 0.008 and 0.03; Figure 4G) and approached statistically significant higher degrees of collagen II expression (*P* = 0.09; Figure 4H). Collagen X mRNA expression was marginally lower with hypoxic differentiation (*P* = 0.06). SOX9 and COMP mRNA expressions and the ratios of collagen II/I and collagen II/X were not significantly different between groups (*P* = 0.20, 0.02, 0.28, and 0.13, respectively). HA scaffolds seeded with BMSCs and differentiated under hypoxia had significantly higher mRNA expressions of aggrecan and collagen II (*P* <0.05; Figure 4G and H) and an increased collagen II/I ratio that approached significance (*P* = 0.09). SOX9, COMP, collagen I, and collagen X mRNA expressions and the ratio of collagen II/X were not significantly different between hypoxic and normoxic differentiation (*P* = 0.62, 0.28, 0.99, 0.31, and 0.13, respectively).

Scaffold composition modulated chondrogenic gene expression of BMSCs. BMSC-seeded collagen scaffolds had notably higher expressions of aggrecan, collagen II, and SOX9 genes than BMSC-seeded HA scaffolds in the majority of oxygen tension groups (*P* <0.05; Figure 4A, D, and F). Within pooled data, BMSCs seeded within collagen scaffolds had augmented aggrecan, collagen II, SOX9, and collagen X gene expressions relative to BMSCs seeded within HA scaffolds regardless of expansion or differentiation oxygen tension (*P* <0.01; Figure 4G, H, and I). Collagen I gene expression was not significantly different between scaffolds under hypoxic expansion, normoxic expansion, or normoxic differentiation (*P* ≥0.18). However, under hypoxic differentiation, collagen I expression was significantly higher in BMSCs seeded within collagen scaffolds relative to HA scaffolds (*P* = 0.03). COMP gene expression was significantly increased in BMSCs seeded on collagen scaffolds only when expansion and differentiation were performed under hypoxia (*P* <0.01).

### Hypoxic isolation/expansion and differentiation enhance chondrogenic extracellular matrix deposition differentially within bone marrow-derived mesenchymal stem cell-seeded collagen and hyaluronic acid scaffolds

**Figure 5:**
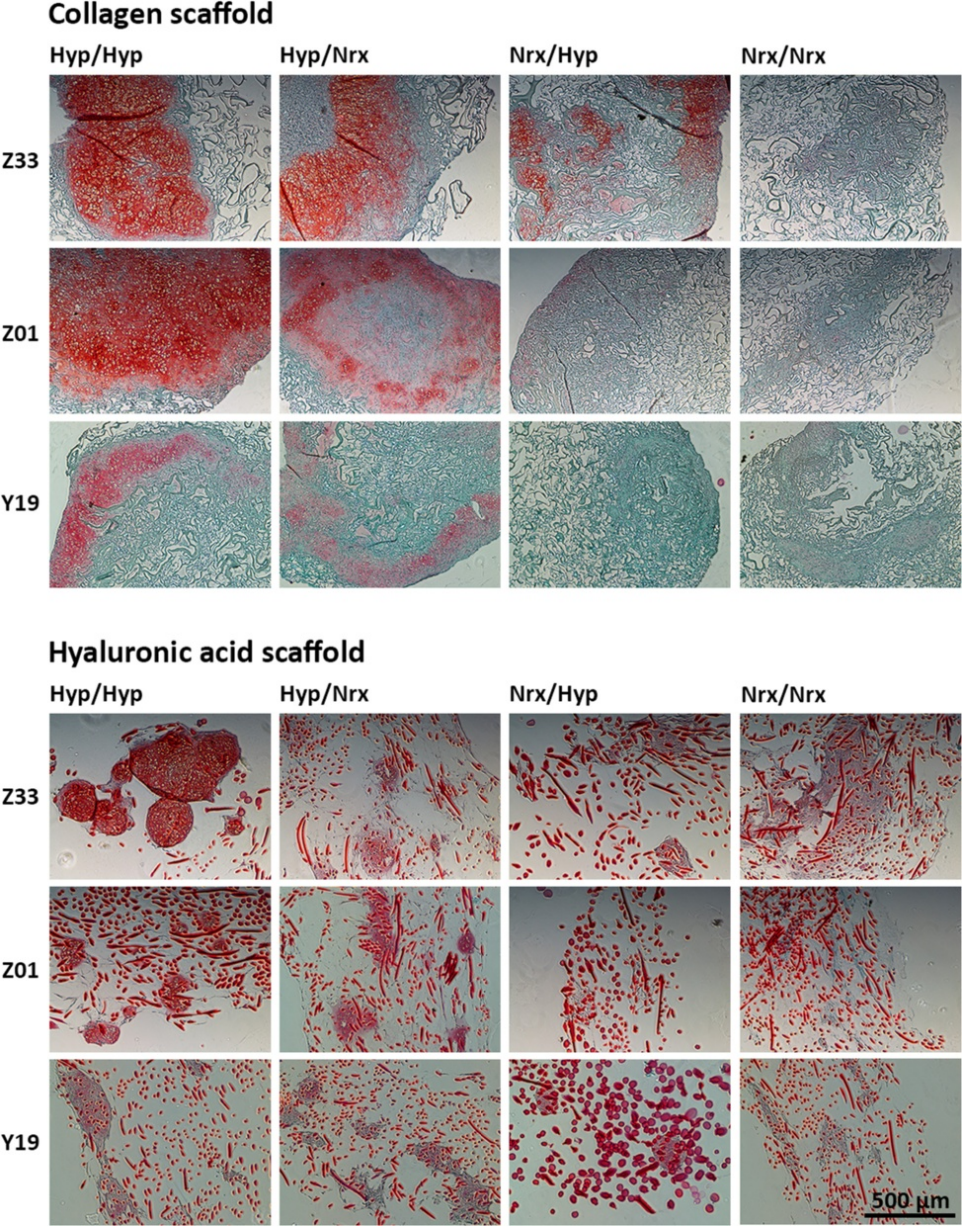
Histological analysis of chondrogenic proteoglycan content within collagen and hyaluronic acid (HA) scaffolds seeded with hypoxia- and normoxia-cultured bone marrow-derived mesenchymal stem cells (BMSCs). BMSCs were isolated/expanded under normoxia or hypoxia, seeded within collagen or HA scaffolds, and subsequently differentiated under normoxia or hypoxia for 14 days in chondrogenic medium. Thereafter, constructs were sectioned at 5-μm thickness and stained with safranin O and fast green. Photomicrographs represent collagen and HA acid scaffolds seeded with BMSCs from three donors.

**Figure 6:**
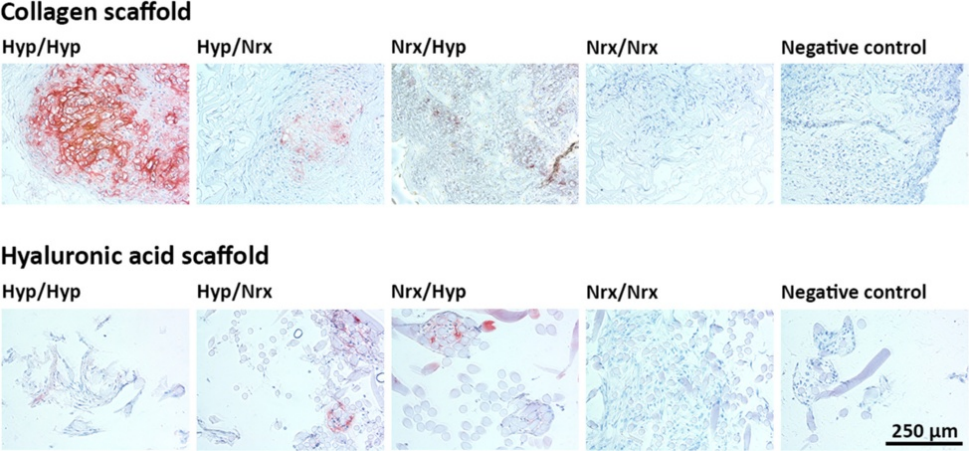
Immunohistochemical analysis of collagen II content within collagen and hyaluronic acid (HA) scaffolds seeded with hypoxia- and normoxia-cultured bone marrow-derived mesenchymal stem cells (BMSCs). BMSCs were isolated/expanded under normoxia or hypoxia, seeded within collagen or HA scaffolds, and subsequently differentiated under normoxia or hypoxia for 14 days in chondrogenic medium. Thereafter, constructs were sectioned at 5-μm thickness, and collagen II immunostaining was performed by using an aminoethylcarbazole-based peroxidase labeling kit. Photomicrographs represent collagen and HA scaffolds seeded with BMSCs from donor Z33.

Safranin O staining was used to assess ECM proteoglycan content within BMSC-seeded collagen and HA scaffolds after 14 days of chondrogenic differentiation (Figure 5). Within collagen scaffolds, ECM was deposited throughout areas of the scaffold with portions containing intense proteoglycan staining noted in constructs that were exposed to hypoxia during the culture period. Widespread staining with safranin O was particularly conspicuous in collagen scaffolds seeded with BMSCs that underwent hypoxic isolation/expansion prior to seeding (Hyp/Hyp and Hyp/Nrx). Hypoxic differentiation also appeared to promote proteoglycan deposition in constructs containing normoxia-expanded BMSCs (Nrx/Hyp). Immunohistochemistry verified the presence of collagen II within constructs that were exposed to hypoxia during isolation/expansion or differentiation (Figure 6). Collagen II content appeared to be most pronounced in constructs exposed to hypoxia throughout the culture period (Hyp/Hyp).

Within HA scaffolds, pockets of ECM were found around HA fibers, which led to the creation of patchy tissue after 14 days of culture (Figure 5). BMSCs that were isolated and expanded under hypoxia demonstrated the most pronounced safranin O staining (Hyp/Hyp and Hyp/Nrx). Although ECM was also noted within HA scaffolds seeded with BMSCs that underwent isolation/expansion under normoxia (Nrx/Hyp and Nrx/Nrx), these constructs lacked safranin O staining. Hypoxic differentiation appeared to augment proteoglycan content in hypoxia-expanded BMSC-seeded HA scaffolds (Hyp/Hyp) but not normoxia-expanded BMSC-seeded HA scaffolds (Nrx/Hyp). Immunohistochemistry revealed the presence of collagen II within ECM in constructs that were exposed to hypoxia during the culture period (Figure 6).

**Figure 7:**
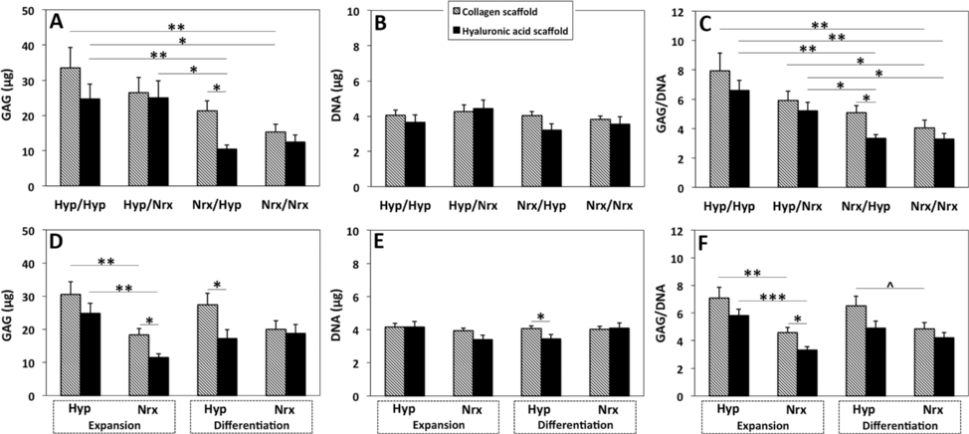
Glycosaminoglycan (GAG) and DNA quantification of collagen and hyaluronic acid (HA) scaffolds seeded with hypoxia- and normoxia-cultured bone marrow-derived mesenchymal stem cells (BMSCs). BMSCs were isolated/expanded under normoxia or hypoxia, seeded within collagen or HA scaffolds, and subsequently differentiated under normoxia or hypoxia for 14 days in chondrogenic medium. **(A**-**C)** Data represent the mean ± standard error of the mean (SEM) of GAG quantity, DNA quantity and GAG/DNA within constructs from five donors in doublets based on oxygen tension group. **(D**-**F)** Data represent mean ± SEM of constructs from the same donors pooled on the basis of oxygen tension during expansion or differentiation. Statistical analysis is represented by the following: unlabeled indicates not significant, ^approaching significance *P* = 0.05-0.10, *significant *P* <0.05, **significant *P* <0.01, and ***significant *P* <0.001.

Quantitative GAG and GAG/DNA values were consistent with histological staining of ECM proteoglycans (Figure 7A and C). DNA quantities were not significantly different between oxygen tension conditions (*P* = 0.23; Figure 7B). GAG production and GAG/DNA in Hyp/Hyp constructs were significantly higher than Nrx/Nrx constructs for BMSCs seeded on both collagen and HA scaffolds (*P* <0.05 and *P* <0.01, respectively; Figure 7A and C). GAG/DNA was also significantly higher in the Hyp/Nrx group than the Nrx/Nrx group regardless of scaffold type (*P* <0.05; Figure 7C). Data pooled on the basis of expansion oxygen showed that hypoxic expansion improved GAG and GAG/DNA regardless of scaffold type (*P* <0.01; Figure 7D and F). Data pooled on the basis of differentiation oxygen tension indicated no significant difference in GAG between hypoxic differentiation and normoxic differentiation for collagen- and HA-based constructs (*P* = 0.12 and 0.45, respectively; Figure 7D). There was a trend for higher GAG/DNA in BMSC-seeded collagen scaffolds differentiated under hypoxia (*P* = 0.09; Figure 7F), and this effect was not seen in BMSC-seeded HA scaffolds (*P* = 0.59).

Scaffold composition had a notable effect on ECM proteoglycan deposition. In a comparison of collagen and HA scaffold-based constructs within each oxygen tension group, a significant difference was seen only in the Nrx/Hyp group in which BMSC-seeded collagen scaffolds had higher GAG and GAG/DNA than BMSC-seeded HA scaffolds (*P* <0.05; Figure 7A and C). Collagen scaffolds that were seeded with normoxia-expanded BMSCs but switched to hypoxic conditions for differentiation (Nrx/Hyp) appeared to have improved GAG/DNA relative to BMSCs that were exposed to sustained normoxic conditions (Nrx/Nrx; *P* = 0.18; Figure 7C). This finding was supported by proteoglycans stained within BMSC-seeded collagen scaffolds (Figure 5). This effect was not seen in BMSC-seeded HA scaffolds, as Nrx/Hyp constructs had stunted GAG/DNA values that were statistically equivalent to Nrx/Nrx constructs (*P* = 0.97) and significantly lower than Hyp/Hyp and Hyp/Nrx constructs (*P* <0.05; Figure 7C). Pooled data showed higher proteoglycan deposition by cells seeded on collagen scaffolds in comparison with HA scaffolds only when expansion was performed under normoxia (*P* = 0.03 and 0.04 for GAG and GAG/DNA, respectively; Figure 7D and F) and when differentiation was performed under hypoxia (*P* = 0.02 for GAG; Figure 7D).

### Oxygen tension and scaffold composition modulate cell-scaffold construct size during chondrogenesis

**Figure 8:**
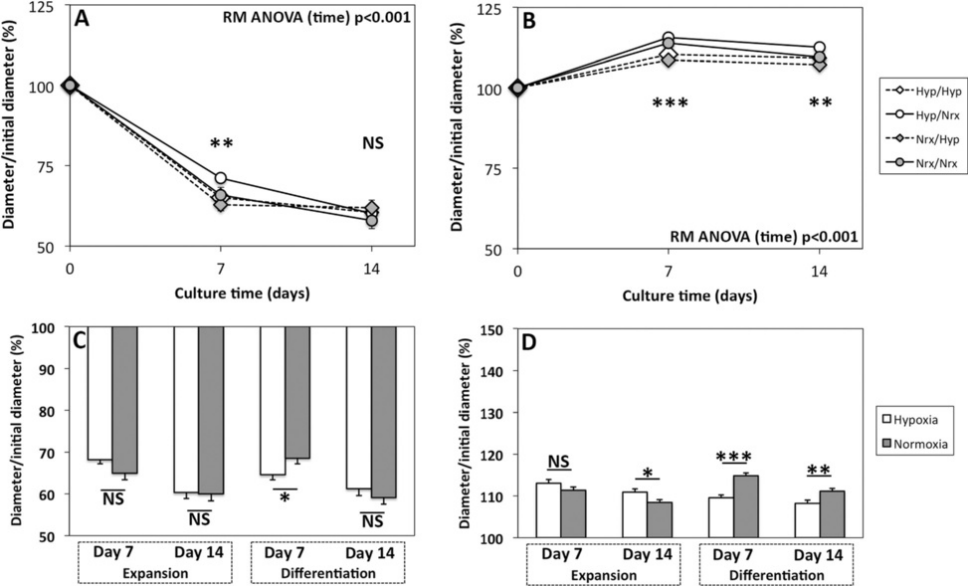
Bone marrow-derived mesenchymal stem cell (BMSC)-seeded scaffold diameter during chondrogenesis. BMSCs were isolated/expanded under normoxia or hypoxia, seeded within collagen or hyaluronic acid (HA) scaffolds, and subsequently differentiated under normoxia or hypoxia for 14 days in chondrogenic medium. Construct diameter was assessed by using high-quality digital photography and ImageJ software. Data represent the mean ± standard error of the mean of constructs containing **(A)** collagen scaffolds or **(B)** HA scaffolds seeded with BMSCs from three donors (five constructs per donor) on the basis of oxygen tension group. Data were pooled on the basis of oxygen tension during expansion or differentiation for **(C)** collagen scaffolds and **(D)** HA scaffold seeded with BMSCs. Statistical analysis is represented by the following: *significant *P* <0.05, **significant *P* <0.01, and ***significant *P* <0.001. NS, not significant; RM ANOVA, repeated measures analysis of variance.

Collagen scaffolds seeded with BMSCs displayed significant diameter contraction with time during chondrogenic culture (*P* <0.001) to 60.1% ± 8.8% of the initial diameter (Figure 8A). A significant difference was noted between oxygen tension groups at day 7 of culture for BMSC-seeded collagen scaffolds (*P* = 0.004) but not at day 14 (*P* = 0.64). Based on pooled analysis, collagen scaffolds seeded with BMSCs expanded under hypoxia had diameters that approached significantly larger values (mean ± SEM of 68.2% ± 1.0%) at day 7 than those seeded with BMSCs expanded under normoxia (65.0% ± 1.6%; *P* = 0.09). This difference was abolished by day 14 with diameters of 60.3% ± 1.5% and 60.0% ± 1.7%, respectively (*P* = 0.88; Figure 8C). BMSC-seeded collagen scaffolds that were differentiated under hypoxia were more contracted (64.6% ± 1.3%) than BMSC-seeded scaffolds differentiated under normoxia at day 7 (68.5% ± 1.4%; *P* = 0.04), but a significant difference was not present by day 14 (61.2% ± 1.7% and 59.1% ± 1.6%; *P* = 0.36).

In contrast to collagen scaffold-based constructs, HA scaffold-based constructs increased in size to 109.7% ± 4.2% of the initial diameter (*P* <0.001; Figure 8B). A significant difference was noted between oxygen tension groups at days 7 and 14 (*P* ≤0.003). Based on pooled analysis, expansion oxygen tension affected HA scaffolds similarly to collagen scaffolds (Figure 8D). Although there was not a significant difference between hypoxia- and normoxia-expanded BMSC-seeded scaffolds at day 7 (113.0% ± 0.8% and 111.3% ± 0.9%, respectively; *P* = 0.15), an effect became statistically significant by day 14 in which hypoxic expansion led to increased diameters relative to normoxic expansion (110.9% ± 0.7% and 108.4% ± 0.8%, respectively; *P* = 0.02). Like BMSC-seeded collagen scaffolds, BMSC-seeded HA scaffolds that were differentiated under hypoxia were more contracted (109.5% ± 0.7%) than BMSC scaffold differentiated under normoxia at day 7 (114.8% ± 0.7%; *P* <0.001). This effect was also seen at day 14 (108.2% ± 0.7% versus 111.1% ± 0.7%; *P* = 0.006).

## Discussion

The major findings of this study are that incubation of ovine BMSCs under hypoxia (3% O_2_) during isolation/expansion and differentiation enhanced *in vitro* chondrogenesis within clinically approved porous collagen I and esterified HA scaffolds and that porous scaffold composition impacted the effect of oxygen tension on chondrogenesis in this *in vitro* model.

Enhancement of chondrogenesis by hypoxic culture was elucidated in BMSC-seeded porous scaffolds, which is an important finding given that cell-seeded scaffolds are used routinely as 3D models of *in vitro* chondrogenesis and clinically in cell transplantation protocols for treatment of cartilage defects [7,21]. It was shown that chondrogenesis could be promoted with hypoxic exposure during distinct isolation/expansion and differentiation conditions. Although oxygen tension during distinct isolation/expansion and differentiation periods was previously studied in detail within pellet and hydrogel models in three studies, the results were inconsistent and warranted further investigation [14,15,19]. In the study at hand, hyaline chondrogenic gene expression of BMSC-seeded porous scaffolds was enhanced with hypoxic exposure during both isolation/expansion and differentiation. This is consistent with our previous findings in a human BMSC pellet model [14]. ECM proteoglycan and collagen II deposition was significantly improved with hypoxic isolation/expansion based on histological and biochemical analyses, whereas the impact of hypoxic differentiation appeared to be less pronounced in this study. Although this finding was demonstrated previously in human BMSC pellets [14], others have reported highly significant findings following hypoxic differentiation in human BMSC pellet [19] and porcine BMSC pellet and hydrogel [15] models. Differences in the effect of hypoxic differentiation between studies could relate to variations in species, duration of incubation, or culture protocols or a combination of these.

BMSC proliferation was also examined, and it was found that hypoxic isolation and expansion to P2 only modestly improved BMSC proliferation. Increased population doublings per day were noted with hypoxic exposure. However, BMSC counts at each passage, cumulative population doublings, and CFU-F colony counts were not significantly different between oxygen tension groups. These results are consistent with previous work that showed non-significant differences during early passages and enhanced proliferation under hypoxia only with prolonged expansion beyond P2 [11,12].

Collagen and HA porous scaffolds were assessed in this study given that these biomaterials are commonly used in cartilage engineering applications and clinical BMSC transplantation [1,2,4,5]. Both scaffolds were capable of fostering gene expression and ECM formation consistent with hyaline-like cartilage from BMSCs cultured *in vitro*. Chondrogenesis on collagen and HA scaffolds was responsive to oxygen tension. However, differences were noted between scaffolds in BMSC gene expression, ECM deposition, and construct size during differentiation. BMSCs seeded and cultured within collagen scaffolds had higher expressions of aggrecan, collagen II, and SOX9 mRNA than BMSCs within HA scaffolds regardless of oxygen tension, which suggests that BMSC differentiation on collagen increases the expression of genes associated with hyaline cartilage relative to the HA. However, BMSCs within collagen scaffolds also had higher mRNA expression of collagen X regardless of oxygen tension and higher expression of collagen I under hypoxic differentiation. These findings suggest that culture on an HA scaffold could modulate gene expression more favorably for hyaline cartilage regeneration by reducing the expression of collagens I and X, which are associated with fibrocartilage and hypertrophic cartilage [29,30] while maintaining the expression of genes associated with hyaline cartilage. Differences between scaffolds were not as pronounced for ECM deposition as gene expression. In most oxygen tension groups, proteoglycan content was not significantly different between collagen- and HA-based constructs. BMSC-seeded collagen scaffolds had higher proteoglycan deposition specifically when expansion was performed under normoxia and differentiation was performed under hypoxia. Hypoxic differentiation appeared to partially reverse dampening of chondrogenesis by normoxic isolation/expansion on BMSC-seeded collagen scaffolds but not on BMSC-seeded HA scaffolds.

The underlying mechanisms for modulation of chondrogenic gene expression and tissue formation by scaffold type were not elucidated in this study but warrant further investigation. BMSCs interact with collagen scaffolds via integrins and with HA scaffolds through CD44 [31,32]. Scaffold characteristics such as stiffness, biomaterial topography, and pore dimension presumably alter intracellular signaling and subsequent processes related to chondrogenesis through these cell surface proteins [30]. Matrix stiffness was found previously to regulate BMSC differentiation and tissue formation [33]. Topographical factors such as scaffold fiber alignment and nanoscale surface features have been shown to influence cell lineage commitment [34,35]. Furthermore, content, size, and orientation of scaffold pores appear to affect BMSC differentiation [36,37]. Porosity alters oxygen diffusion through scaffolds, which could also modulate chondrogenesis through the creation of oxygen gradients [38].

BMSC-scaffold construct diameter was investigated during culture given that fluctuations in construct size occur with tissue formation and remodeling and ultimately impact the suitability for constructs for implantation *in vivo*. Size was maintained during culture in BMSC-seeded HA scaffolds, whereas BMSC-seeded collagen scaffolds exhibited progressive contraction with time. Variation in size presumably involves a balance of cell-scaffold interactions, biomaterial chemistry, scaffold degradation, and ECM formation. Cell-mediated contraction that occurs through smooth muscle actin has been described in detail within collagen-based constructs [39]. HA is hydrophilic and may promote increased scaffold swelling through water absorption relative to collagen I [23]. Biomaterial degradation, hydrolysis, or fragmentation may reduce the size of a construct if an adequate amount of ECM has not been deposited by the time that these processes occur [23,40,41]. In the study at hand, it is not known how scaffold size affected chondrogenesis, although collagen scaffold contraction presumably led to increased cell density per volume of scaffold, which could have modulated cell-cell interactions and increased chondrogenesis [42]. It is also possible that contraction inhibited chondrogenesis, as it was shown previously that contraction may promote differentiation to a fibroblastic lineage [43,44]. Given that esterified HA scaffold size was maintained, the findings of this study suggest that, in a clinical setting, implantation of BMSCs on an esterified HA scaffold could promote better early cartilage defect filling than on a collagen scaffold.

Construct size was also affected by incubator oxygen tension. Both collagen and HA scaffold seeded with BMSCs had increased diameters when BMSC isolation and expansion were performed under hypoxia and differentiation was performed under normoxia. Various mechanisms could be involved: modulation of cell-mediated contraction through smooth muscle actin and cytoskeletal alteration [39], ECM proteoglycan formation [14,45], crosslinking via lysyl oxidase [46], or degradation through matrix metalloproteinases [47].

This study has some limitations. Although two defining criteria of mesenchymal stem cells—plastic adherence and multipotential differentiation—were confirmed, determination of cell surface antigens was not performed to fulfill the third criterion of the Mesenchymal and Tissue Stem Cell Committee [28]. This third criterion was omitted because the study at hand involved an identical method of BMSC isolation and expansion described in our previous work, which included flow cytometric analysis of cell surface markers [14]. Secondly, the findings of this study demonstrate enhanced *in vitro* chondrogenesis with hypoxic culture, although it is still not clear whether *in vitro* hypoxic culture improves chondrogenesis following implantation within cartilage defects and prolonged *in vivo* exposure to joint elements. Joints have been shown to be hypoxic, and it is possible that this characteristic could promote *in vivo* chondrogenesis regardless of the *in vitro* culture conditions prior to construct implantation [10]. Lastly, ovine rather than human BMSCs were used in this study. Although sheep are routinely used as animal models in cartilage engineering studies [13,48,49], differences between species could impact the clinical applicability of our findings.

## Conclusions

BMSCs seeded on clinically relevant collagen I and esterified HA porous scaffolds displayed enhanced *in vitro* chondrogenesis with hypoxic incubation during distinct isolation/expansion and differentiation culture periods. Hypoxic culture of BMSCs may therefore play a role in improving cartilaginous tissue formation following transplantation of BMSC-seeded scaffolds. Both collagen I and esterified HA scaffold supported the creation of hyaline-like engineered cartilage. However, differences were noted in chondrogenic gene expression, ECM deposition, and cell-scaffold construct size during differentiation that could impact the choice of biomaterial for use in BMSC transplantation protocols.

## Additional file

Additional file 1:
**DNA sequencing of reverse transcription-quantitative real-time polymerase chain reaction (RT-qPCR) products.** BLAST, Basic Local Alignment Search Tool; NCBI, National Center for Biotechnology Information.

## Abbreviations

3D: three-dimensional
α-MEM: alpha-minimal essential medium
ANOVA: analysis of variance
BMSC: bone marrow-derived mesenchymal stem cell
CFU-F: colony-forming unit fibroblastic
COMP: cartilage oligomeric matrix protein
DMEM: Dulbecco’s modified Eagle’s medium
ECM: extracellular matrix
EDTA: ethylenediaminetetraacetic acid
FBS: fetal bovine serum
FGF-2: fibroblast growth factor-2
GAG: glycosaminoglycan
HA: hyaluronic acid
HEPES: 4-(2-hydroxyethyl)-1-piperazineethanesulfonic acid
ITS: insulin-transferrin-selenium
MNC: mononucleated cell
NCBI: National Center for Biotechnology Information
P: passage
PBS: phosphate-buffered saline
RT-qPCR: reverse transcription-quantitative real-time polymerase chain reaction
SEM: standard error of the mean
SOX9: sex-determining region Y (SRY)-box 9
