# Supplementary material for: Hypoxic culture of bone marrow-derived mesenchymal stromal stem cells differentially enhances in vitro chondrogenesis within cell-seeded collagen and hyaluronic acid porous scaffolds
Source: Stem Cell Res Ther. 2015 Apr 23;6(1):84. doi: 10.1186/s13287-015-0075-4 (PMC4431536; doi:10.1186/s13287-015-0075-4)
Supplement: Additional file 1: — DNA sequencing of reverse transcription-quantitative real-time polymerase chain reaction (RT-qPCR) products. BLAST, Basic Local Alignment Search Tool; NCBI, National Center for Biotechnology Information. [file 13287_2015_75_MOESM1_ESM.pdf]

Beta-actin (ACTB)

Forward primer
5’-cggcgggaccaccat-3’ NCBI sequence base pairs: 986-1000

Reverse primer
5’-gcagtgatctctttctgcatcct-3’ NCBI sequence base pairs: 1020-1042

PCR product within NCBI sequence
Ovis aries actin, beta (ACTB), mRNA – starting at base pair 981

```
5′-ctgtccggcg ggaccaccat gtaccctggc atcgcagaca ggatgcagaa agagatcact gccctg-3′
3′-gacaggccgc cctggtggta catgggaccg tagcgtctgt cctacgtctt tctctagtga cgggac-5′
```

Sanger sequencing of PCR product using forward primer
Clearly sequenced region on chromatograph: 5’-gaaagagatcactgc-3’
BLAST of sequenced region: Ovis aries actin, beta (ACTB), mRNA (100% coverage, 100% identification)

```
5′-ctgtccggcg ggaccaccat gtaccctggc atcgcagaca ggatgcagaa agagatcact gccctg-3′
3′-gacaggccgc cctggtggta catgggaccg tagcgtctgt cctacgtctt tctctagtga cgggac-5′
```

Sanger sequencing of PCR product using reverse primer
Clearly sequenced region on chromatograph: 5’-catggtggtcccgccg-3’
BLAST of sequenced region: Ovis aries actin, beta (ACTB), mRNA (100% coverage, 100% identification)

```
5′-ctgtccggcg ggaccaccat gtaccctggc atcgcagaca ggatgcagaa agagatcact gccctg-3′
3′-gacaggccgc cctggtggta catgggaccg tagcgtctgt cctacgtctt tctctagtga cgggac-5′
```

Aggrecan (ACAN)

Forward primer

5'-tggaatgatgtcccatgcaa-3'   NCBI sequence base pairs: 6019-6038

Reverse primer

5'-gccactgtgcccttttttacag-3'   NCBI sequence base pairs: 6077-6057

PCR product within NCBI sequence

Ovis aries aggrecan (ACAN), mRNA (predicted) - starting at base pair 6015

```
5'-cgagtg gaatgatgtc ccatgcaatt accagctgcc cttcacctgt aaaaagggca cagtggcctg-3'
3'-gctcac cttactacag ggtacgttaa tggtcgacgg gaagtggaca tttttcccgt gtcaccggac-5'
```

Sanger sequencing of PCR product using forward primer

Clearly sequenced region on chromatograph: 5'-cccttcacctgtaaaaagggcacagtggc-3'

BLAST of sequenced region: Ovis aries aggrecan (ACAN), mRNA (100% coverage, 100% identification)

```
5'-cgagtg gaatgatgtc ccatgcaatt accagctgcc cttcacctgt aaaaagggca cagtggcctg-3'
3'-gctcac cttactacag ggtacgttaa tggtcgacgg gaagtggaca tttttcccgt gtcaccggac-5'
```

Sanger sequencing of PCR product using reverse primer

Clearly sequenced region on chromatograph: 5'-ctggtaattgcatgggacatcattcca-3'

BLAST of sequenced region: Ovis aries aggrecan (ACAN), mRNA (100% coverage, 100% identification)

```
5'-cgagtg gaatgatgtc ccatgcaatt accagctgcc cttcacctgt aaaaagggca cagtggcctg-3'
3'-gctcac cttactacag ggtacgttaa tggtcgacgg gaagtggaca tttttcccgt gtcaccggac-5'
```

Cartilage oligomeric matrix protein (COMP)

Forward primer
5'-cctaactgggtggtgctcaac-3'   NCBI sequence base pairs: 1654-1674

Reverse primer
5'-ctgggtcgctgttcatcgt-3'   NCBI sequence base pairs: 1714-1696

PCR product within NCBI sequence
Ovis aries cartilage oligomeric matrix protein (COMP), mRNA (predicted) - starting at base pair 1651

```
5'-gaccctaact gggtggtgct caaccagggt atggagatcg tgcagacgat gaacagcgac ccaggcct-3'
3'-ctgggattga cccaccacga gttggtccca tacctctagc acgtctgcta cttgtcgctg ggtccgga-5'
```

Sanger sequencing of PCR product using forward primer
Clearly sequenced region on chromatograph: 5'-tgcagacgatgaacagcgacccag-3'
BLAST of sequenced region: Ovis aries cartilage oligomeric matrix protein (COMP), mRNA (100% coverage and 100% identification)

```
5'-gaccctaact gggtggtgct caaccagggt atggagatcg tgcagacgat gaacagcgac ccaggcct-3'
3'-ctgggattga cccaccacga gttggtccca tacctctagc acgtctgcta cttgtcgctg ggtccgga-5'
```

Sanger sequencing of PCR product using reverse primer
Clearly sequenced region on chromatograph: 5'-ccctggttgagcaccacccagttagg-3'
BLAST of sequenced region: Ovis aries cartilage oligomeric matrix protein (COMP), mRNA (100% coverage and 100% identification)

```
5'-gaccctaact gggtggtgct caaccagggt atggagatcg tgcagacgat gaacagcgac ccaggcct-3'
3'-ctgggattga cccaccacga gttggtccca tacctctagc acgtctgcta cttgtcgctg ggtccgga-5'
```

Collagen I (COL1A1)

Forward primer
5'-cgccccagaccaggaatt-3'   NCBI sequence base pairs: 4419-4436

Reverse primer
5'-gtggaaggagtttacaggaagca-3'   NCBI sequence base pairs: 4481-4459

PCR product within NCBI sequence
Ovis aries collagen, type I, alpha 1 (COL1A1), mRNA (predicted) - starting at base pair 4415

```
5'-ttggcg ccccagacca ggaattcggc ttcgacatcg gctctgtctg cttcctgtaa actccttcca cccc-3'
3'-aaccgc ggggtctggt ccttaagccg aagctgtagc cgagacagac gaaggacatt tgaggaaggt gggg-5'
```

Sanger sequencing of PCR product using forward primer
Clearly sequenced region on chromatograph: 5'-gtctgcttcctgtaaactccttccac-3'
BLAST of sequenced region: Ovis aries collagen, type I, alpha 1 (COL1A1), mRNA (100% coverage and 100% identification)

```
5'-ttggcg ccccagacca ggaattcggc ttcgacatcg gctctgtctg cttcctgtaa actccttcca cccc-3'
3'-aaccgc ggggtctggt ccttaagccg aagctgtagc cgagacagac gaaggacatt tgaggaaggt gggg-5'
```

Sanger sequencing of PCR product using reverse primer
Clearly sequenced region on chromatograph: 5'-tcgaagccgaattcctggtctggggcg-3'
BLAST of sequenced region: Ovis aries collagen, type I, alpha 1 (COL1A1), mRNA (100% coverage and 100% identification)

```
5'-ttggcg ccccagacca ggaattcggc ttcgacatcg gctctgtctg cttcctgtaa actccttcca cccc-3'
3'-aaccgc ggggtctggt ccttaagccg aagctgtagc cgagacagac gaaggacatt tgaggaaggt gggg-5'
```

Collagen II (COL2A1)

Forward primer
5'-gacctcacgtctccccatca-3'   NCBI sequence base pairs: 4371-4390

Reverse primer
5'-ctgctcgggccctcctat-3'   NCBI sequence base pairs: 4428-4411

PCR product within NCBI sequence
Ovis aries collagen, type II, alpha 1 (COL2A1), mRNA (predicted) - starting at base pair 4371

```
5'-gacctcacgt ctccccatca ttgacattgc acccatggac ataggagggc ccgagcagga attc-3'
3'-ctggagtgca gaggggtagt aactgtaacg tgggtacctg tatcctcccg ggctcgtcct taag-5'
```

Sanger sequencing of PCR product using forward primer
Clearly sequenced region on chromatograph: 5'-tggacataggagggcccgagcag-3'
BLAST of sequenced region: Ovis aries collagen, type II, alpha 1 (COL2A1), mRNA (100% coverage and 100% identification)

```
5'-gacctcacgt ctccccatca ttgacattgc acccatggac ataggagggc ccgagcagga attc-3'
3'-ctggagtgca gaggggtagt aactgtaacg tgggtacctg tatcctcccg ggctcgtcct taag-5'
```

Sanger sequencing of PCR product using reverse primer
Clearly sequenced region on chromatograph: 5'-caatgatggggagacgtgaggt-3'
BLAST of sequenced region: Ovis aries collagen, type II, alpha 1 (COL2A1), mRNA (100% coverage and 100% identification)

```
5'-gacctcacgt ctccccatca ttgacattgc acccatggac ataggagggc ccgagcagga attcg-3'
3'-ctggagtgca gaggggtagt aactgtaacg tgggtacctg tatcctcccg ggctcgtcct taagc-5'
```

Collagen X (COL10A1)

Forward primer
5'-caggctcgaatgggctgtac-3' NCBI sequence base pairs: 2000-2019

Reverse primer
5'-ccaccaagaatcctgagaaagag-3' NCBI sequence base pairs: 2062-2040

PCR product within NCBI sequence
Ovis aries collagen, type X, alpha 1 (COL10A1), mRNA (predicted) - starting at base pair 1996

```
5'-aatgc aggctcgaat gggctgtact cctctgagta cgtccactcc tctttctcag gattcttggt ggctcc-3'
3'-ttacg tccgagctta cccgacatga ggagactcat gcaggtgagg agaaagagtc ctaagaacca ccgagg-5'
```

Sanger sequencing of PCR product using forward primer
Clearly sequenced region on chromatograph: 5'-ccactcctctttctcaggattcttggtgg-3'
BLAST of sequenced region: Ovis aries collagen, type X, alpha 1 (COL10A1), mRNA (100% coverage and 100% identification)

```
5'-aatgc aggctcgaat gggctgtact cctctgagta cgtccactcc tctttctcag gattcttggt ggctcc-3'
3'-ttacg tccgagctta cccgacatga ggagactcat gcaggtgagg agaaagagtc ctaagaacca ccgagg-5'
```

Sanger sequencing of PCR product using reverse primer
Clearly sequenced region on chromatograph: 5'-ctcagaggagtacagcccattcgagcctg-3'
BLAST of sequenced region: Ovis aries collagen, type X, alpha 1 (COL10A1), mRNA (100% coverage and 100% identification)

```
5'-aatgc aggctcgaat gggctgtact cctctgagta cgtccactcc tctttctcag gattcttggt ggctcc-3'
3'-ttacg tccgagctta cccgacatga ggagactcat gcaggtgagg agaaagagtc ctaagaacca ccgagg-5'
```

Sex determining region Y-box 9 (SOX9)

Forward primer

5'-gctgctggccgtgatga-3' NCBI sequence base pairs: 956-972

Reverse primer

5'-gggtcgcgcgtttgttt-3' NCBI sequence base pairs: 1007-995

PCR product within NCBI sequence

Ovis aries SRY (sex determining region Y)-box 9 (SOX9), mRNA (predicted) - starting at base pair 951

```
5'-cccacgctgc tggccgtgat gatcgcagaa agaacccaag aaacaaacgc gcgacccttt-3'
3'-gggtgcgacg accggcacta ctagcgtctt tcttgggttc tttgtttgcg cgctgggaaa-5'
```

Sanger sequencing of PCR product using forward primer

Clearly sequenced region on chromatograph: 5'-aaacgcgcgaccc-3'

BLAST of sequenced region: Ovis aries SRY (sex determining region Y)-box 9 (SOX9), mRNA (100% coverage and 100% identification)

```
5'-cccacgctgc tggccgtgat gatcgcagaa agaacccaag aaacaaacgc gcgacccttt-3'
3'-gggtgcgacg accggcacta ctagcgtctt tcttgggttc tttgtttgcg cgctgggaaa-5'
```

Sanger sequencing of PCR product using reverse primer

Clearly sequenced region on chromatograph: 5'-catcacggccagcagc-3'

BLAST of sequenced region: Ovis aries SRY (sex determining region Y)-box 9 (SOX9), mRNA (100% coverage and 100% identification)

```
5'-cccacgctgc tggccgtgat gatcgcagaa agaacccaag aaacaaacgc gcgacccttt-3'
3'-gggtgcgacg accggcacta ctagcgtctt tcttgggttc tttgtttgcg cgctgggaaa-5'
```
